# Factors associated with skipping breakfast among Inner Mongolia Medical students in China

**DOI:** 10.1186/1471-2458-13-42

**Published:** 2013-01-17

**Authors:** Juan Sun, He Yi, Zhiyue Liu, Yan Wu, Jiang Bian, Yanyan Wu, Yuki Eshita, Gaimei Li, Qing Zhang, Ying Yang

**Affiliations:** 1 Inner Mongolia Medical College, Inner Mongolia Minority Autonomous Region, China; 2 Oita University, Faculty of Medicine, Oita, Japan; 3 Inner Mongolia Normal University Institute of Media, Inner Mongolia Minority Autonomous Region, China

**Keywords:** Breakfast, Behavior, Medical students, Prevalence

## Abstract

**Background:**

Few studies on the breakfast consumption habits of medical students in China have been carried out. The aim of the present study was to determine the prevalence of skipping breakfast and factors associated with skipping breakfast among medical students in Inner Mongolia of China, and to assist in the design of interventions to improve breakfast consumption habits of medical college students in this region.

**Methods:**

From December 2010 to January 2011 a cross-sectional survey was conducted among medical students in the Inner Mongolia Medical College using a self-administered questionnaire. The prevalence of skipping breakfast in relation to lifestyle habits was described and factors associated with breakfast consumption were identified using multiple logistic regression analysis.

**Results:**

The overall prevalence of skipping breakfast was 41.7% and 23.5% for males and females, respectively. The Faculty of Medicine Information Management had the highest breakfast skipping prevalence. Logistic regression models found that the main factors associated with breakfast consumption habits among medical students were gender, class years of education, monthly expenses, faculty, appetite, sleeping quality, and the learning process; monthly expenses, sleeping quality, and the learning process showed a dose-dependent relationship.

**Conclusions:**

Breakfast consumption was associated with many factors, most importantly monthly expenses, sleeping quality and the learning process. The prevalence of skipping breakfast is significantly higher compared recently reported figures for medical students in western countries and other areas of China. Improvement of breakfast education should be considered for students in which higher monthly expenses, poor sleeping quality, or a laborious learning process have been identified.

## Background

The provision of energy and nutrients throughout the day is extremely important and breakfast has been considered an important dietary factor for energy regulation [1]. Moreover, in the 1980s, some studies reported that breakfast could play an important role in the prevention of adult chronic diseases such as heart disease, cancer, diabetes, and osteoporosis [2]. In addition, for university students, breakfast consumption is associated with a range of positive outcomes, including better school attendance, academic performance, nutrient intake, fitness, and appropriate body weight [3-5].

Missing breakfast on the other hand has been associated with adverse effects on cognitive function (including memory), academic performance, school attendance, psychosocial function, and mood in children and young people [6]. Furthermore, reduced breakfast energy intake is associated with higher total daily energy intake [7] and when breakfast is skipped it can be difficult to properly compensate for it later in the day. For example, those people who skipped breakfast are reported to have higher daily intakes of fat, cholesterol, and energy, and lower intakes of fiber, vitamins, and minerals in comparison to breakfast eaters, thereby increasing the likelihood of gastrointestinal disease later in life [8].

Adolescence is a time of transition and often results in a weakening of dietary habits over a period of time concurrent with the search for independence [9]. In China, university students often pay insufficient attention to breakfast, with some individuals missing breakfast because it takes too much time [10].

Because health personnel are important promoters and role models for maintaining a healthy lifestyle for the general population [11], early in 1999 the World Health Organization (WHO) endorsed the position that physicians, as role models of healthy living, should not overlook their own lifestyle habits. From the educator’s perspective university and college arenas represent the final opportunity for health and nutritional education of a large number of medical students with the object of ensuring the development of a healthy lifestyle that can be subsequently promoted after they graduate and enter the health care profession [12,13].

Many studies of breakfast consumption were carried out in the 1980s by different research groups to examine the type of food consumed around the world [2,14,15]. In this century, there has been more in-depth study and focus on breakfast [16,17]. In China, research has been carried out in different populations to study breakfast consumption habits, type of food consumed, and the nutrients supplied. Although sample sizes were small, it was noted that poor dietary habits were part of this complex issue [18], and that in different regions, breakfast consumption habits varied considerably [19].

Inner Mongolia is a region inhabited by the Mongolian ethnic minority although considerable numbers of Han Chinese also reside there. The current survey was conducted among medical students of the Inner Mongolia Medical College with the aim of documenting the prevalence of skipping breakfast. A longer-term goal was to use the survey results to assist with design of interventions to change breakfast consumption habit*s* to ensure enough energy and nutrient intake in the morning among medical college students.

## Methods

### Setting and target population

A cross-sectional survey was conducted among medical students at the Inner Mongolia Medical College of China the methodology of which has been described in a previous study [20]. The survey focused on medical students and employed a self-administered questionnaire.

A total 6044 students from the Inner Mongolia Medical College campus completed the questionnaire. All participants came from the faculties of Clinical Medicine, Public Health Administration, Medicine Information Management, Medicine, Traditional Chinese Medicine, or Mongolian Medicine. Clinical Medical students have internships in a hospital for the last 2 years of the program, which is off campus. Consequently, for this faculty we only surveyed students in years 1–3 of their education.

### Sampling procedure

All medical students from Inner Mongolia Medical College campus were invited to participate and students completed the questionnaires in the classroom. A member of our study group explained the purpose of the survey, and the privacy protection policy for personal and enrollment data, and checked completed questionnaires. Participants returned the completed anonymous self-administered questionnaire in a sealed envelope to members of our study group.

### Questionnaire and measures

The questionnaire comprised two sections. The first section contained inquiries about basic demographic data, which including sex, age, ethnicity, residence, and faculty while the second contained questions regarding breakfast consumption habits of the participants during time at college.

We defined breakfast per the Student Nutrition Dietary Assessment, which is any food or beverage consumption between awakening and 45 minutes after the start of school [15,21]. Subjects who did not consume breakfast on one of two days or neither day were categorized as breakfast skippers, while those that consumed breakfast on both days were classified as breakfast eaters [22].

Sleeping quality was assessed through a question describing self-sensation, with possible responses (3 levels, good, medium, and bad) varying from good (feeling energetic after getting up) to bad (feeling befuddled or in a bad mood after getting up). Physical condition was evaluated similarly, with possible responses of good (able to be effective [no problems] in work and leisure activities) to bad (unable to be effective in work and leisure activities). For appetite evaluation, extreme response alternatives were good (want to eat when it is time to eat to bad loss of appetite time to eat).

Ethnicity was categorized as Han, Mongolian, or Other.

### Statistical analysis

The prevalence of skipping breakfast was determined from one survey item and in relation to demographic characteristics and some lifestyle habits. Multiple logistic regression analysis was used to ascertain factors associated with breakfast eating. In multiple logistic regression analysis, eating breakfast daily was considered the dependent variable, and the following as independent variables: gender, ethnicity, class years of education, monthly expenses, residence type, faculty, physical condition, relationships, appetite, sleeping quality, the learning process, and getting along with classmates.

We calculated crude odds ratios (OR) to evaluate the risk of independent variables and associated 95% confidence intervals. We then used multivariate logistic regression models to adjust for the possible confounding influences between the independent variables on the dependent in model. We selected all the multilevel variables that were significant in the multivariate logistic regression and used the linear-by-linear association chi square test to evaluate each factor’s linear trend.

Age and class years of education (5 levels) were expressed as the mean and standard deviation (SD). Statistical comparisons employed ANOVA with the (Student-Newman-Keuls) post hoc test to evaluate the differences in each class year of education. We calculated the linear correlation between average age and class years of education.

Quantitative data were inputted using EpiDate (Version 3.1), then transferred into SPSS (Version 13.0). All statistical analyses were performed using SPSS for Windows Version 13.0, with a significance level of P < 0.05.

### Ethical approval

Ethical approval to conduct the study was obtained from the Ethical Committee of Inner Mongolia.

## Results

### Participant characteristics

Six students did not answer the question about eating breakfast, leaving data for 6038 students available for analysis: 1772 were male (29.3%) and 4266 were female (70.7%). The response prevalence was 99.9%.

The mean age of the participants by year of study was: year 1: 20.16 years (SD=1.16); year 2: 21.19 years (SD=1.07); year 3: 22.29 years (SD=1.12) year 4: 23.25 years (SD=1.12); and year 5: 24.02 years (SD=1.19), respectively. Mean age was significantly different by class year (P< 0.001). There was a linear increase in students’ mean age with years of study 1 to 5 with mean age increasing about 1 year as year of study increased by 1 year. Consequently, similar to our previous study [17], class years of education was a good proxy for age and thus we used the class years of education throughout our study.

### Prevalence of breakfast consumption

The prevalence of skipping breakfast consumption was 28.9%. The prevalence of skipping breakfast consumption in relation to lifestyle habits is shown in Table 1. Skipping breakfast consumption prevalence among male students was significantly higher compared to female students (41.7% vs. 23.5%). The lowest prevalence of skipping breakfast consumption was found among medical students belonging to the faculties of Traditional Chinese Medicine and Mongolian Medicine (22.7%), and was significantly lower among students whose the monthly expenses were <300 yuan compared to students whose monthly expenses were >1000 yuan (19.7% vs. 46.1%). The prevalence of skipping breakfast among male students in their second year of education was 49.7%, increasing to 54.3% for male students in their fourth year of education. The number of breakfast skippers was about 30%, in the second, third, and fourth year while it was lower in the first and the last year.

**Table 1:** Breakfast consumption prevalence among students according to demographic characteristics

**Category**	**n**	**%**
**Total**	6038	28.9%
**Gender (n=6038)**		
Male	1772	41.7%
Female	4266	23.5%
**Ethnicity (n=6038)**		
Han ethnicity	4312	28.5%
Mongolian ethnicity	1405	28.5%
Other ethnicity	321	34.9%
**Class years of education (n=6038)**		
1	2471	23.2%
2	1925	34.1%
3	1272	30.7%
4	284	37.7%
5	86	17.4%
**Monthly expenses (n=6022)**		
<300	467	19.7%
300-600	3181	24.7%
600-1000	2081	34.9%
>1000	293	46.1%
**Residence (n=6019)**		
City	2249	30.0%
Rural	3341	28.1%
Pastoral	195	24.1%
Suburbs	234	32.1%
**Faculty (n=6038)**		
Clinical Medicine	2191	26.9%
Public Administration and Information Management	800	35.0%
Medicine	949	35.0%
Traditional Chinese Medicine and Mongolian Medicine	1023	22.7%
Other	1075	28.7%

### Self-perception and breakfast consumption prevalence

Breakfast consumption prevalence among students with good self-perception about learning was significantly higher than for students with bad self-perception (81.4% vs. 51.2%; Table 2); it was also over 1.5 times higher among students with good self-perception about appetite compared to students with bad self-perception about appetite. The prevalence of eating breakfast regularly in medical students having good self-perception about sleeping quality was significantly higher than students with bad self-perception about sleeping quality (75.8% vs. 57.9%).

**Table 2:** Students’ self-perception of breakfast consumption associated factors

**Category**	**n**	**%**
**Physical condition (n=6024)**		
Good	3589	74.3%
Medium	2238	66.8%
Bad	197	61.4%
**Relationships (n=6020)**		
Good	2421	73.1%
Medium	3464	69.8%
Bad	135	69.6%
**Appetite (n=6020)**		
Good	3582	77.0%
Medium	2146	64.4%
Bad	292	49.3%
**Sleeping (n=6019)**		
Good	3216	75.8%
Medium	2266	67.6%
Bad	537	57.9%
**Learning (n=6008)**		
Good	1255	81.4%
Medium	4253	70.6%
Bad	500	51.2%
**Get along with classmates (n=6002)**		
Good	3682	73.7%
Medium	2266	67.1%
Bad	54	64.8%

### Factors associated with breakfast consumption

Breakfast consumption prevalence among different ethnic groups was not significantly different (Table 3). However, compared with the first class year of education, medical students in the second to fourth class years of education were less likely to eat breakfast regularly. Monthly expenses were also strongly and negatively associated with breakfast consumption habits. Students in the faculties of Traditional Chinese Medicine and Mongolian Medicine were more likely to eat breakfast compared with students in other faculties. Finally, multiple logistic regression analysis clearly showed that better sleeping quality is a protective factor in regard to medical students’ breakfast consumption habits.

**Table 3:** Logistic regression analysis of regular breakfast consumption among medical students

**Category**	**Crude OR**	**95%CL**	**P**	**Adjusted OR**	**95%CL**	**P**
**Gender (n=6038)**			<0.01			<0.01
Male	1.00	reference		1.00	reference	
Female	0.43	0.38-0.48		2.20	1.93-2.506	
**Ethnicity (n=6038)**			0.05			0.23
Han ethnicity	1.00	reference		1.00	reference	
Mongolian ethnicity	1.34	1.06-1.71		0.90	0.78-1.05	
Other ethnicity	1.35	1.04-1.74		0.85	0.66-1.10	
**Class years of education (n=6038)**			<0.01			<0.01
1	1.00	reference		1.00	reference	
2	0.58	0.51-0.67		0.68	0.58-0.79	
3	0.68	0.59-0.79		0.78	0.66-0.94	
4	0.50	0.39-0.65		0.68	0.50-0.91	
5	1.43	0.81-2.51		1.34	0.74-2.43	
**Monthly expenses (n=6022)**			<0.01			<0.01
<300	1.00	reference		1.00	reference	
300-600	0.75	0.59-0.95		0.82	0.64-1.06	
600-1000	0.46	0.36-0.59		0.56	0.43-0.72	
>1000	0.29	0.21-0.4		0.36	0.25-0.51	
**Residence (n=6019)**			0.14			0.33
City	1.00	reference		1.00	reference	
Rural	1.10	0.97-1.23		0.88	0.77-1.01	
Pastoral	1.35	0.96-1.9		0.99	0.69-1.45	
Suburbs	0.90	0.68-1.21		0.96	0.70-1.30	
**faculty (n=6038)**			<0.01			<0.01
Clinical Medicine	1.00	reference		1.00	reference	
Public Administration and Information Management	0.68	0.57-0.81		0.72	0.60-0.87	
Medicine	0.68	0.58-0.8		0.70	0.58-0.84	
Traditional Chinese Medicine and Mongolian Medicine	1.25	1.05-1.49		1.30	1.08-1.59	
Other	0.91	0.78-1.08		0.61	0.51-0.74	
**Physical condition (n=6024)**			<0.01			0.28
Good	1.00	reference		1.00	reference	
Medium	0.67	0.62-0.78		0.90	0.79-1.03	
Bad	0.55	0.41-0.74		0.99	0.69-1.45	
**Relationships (n=6020)**			0.22			0.26
Good	1.00	reference		1.00	reference	
Medium	0.85	0.76-0.396		1.08	0.93-1.24	
Bad	0.84	0.58-1.231		1.40	0.91-2.16	
**Appetite (n=6020)**			<0.01			<0.01
Good	1.00	reference		1.00	reference	
Medium	0.54	0.48-0.61		0.64	0.56-0.74	
Bad	0.29	0.23-0.37		0.44	0.33-0.57	
**Sleeping (n=6019)**			<0.01			<0.01
Good	1.00	reference		1.00	reference	
Medium	0.67	0.59-0.75		0.89	0.78-1.02	
Bad	0.44	0.36-0.53		0.70	0.56-0.86	
**Learning (n=6008)**			<0.01			<0.01
Good	1.00	reference		1.00	reference	
Medium	0.55	0.47-0.64		0.67	0.56-0.80	
Bad	0.24	0.19-0.3		0.40	0.31-0.52	
**Get along with classmates (n=6002)**			<0.01			0.18
Good	1.00	reference		1.00	reference	
Medium	0.73	0.65-0.82		0.88	0.76-1.01	
Bad	0.66	0.38-1.16		0.91	0.50-1.68	

### Linear-by-linear association chi-square test

The results of the linear-by-linear association chi square test for multilevel variables showed that with the exception of class years of education, monthly expenses, sleeping quality, and the learning process presented a dose response relationship (Table 4).

**Table 4:** Linear-by-linear association chi-square test

**Category**	***Value***	***P***
**Class years of education**	0.32	0.57
**Monthly expenses**	120.52	>0.01
**Sleeping quality**	93.34	>0.01
**Learning process**	152.84	>0.01

## Discussion

Eating breakfast containing adequate levels of tryptophan are an important mechanism by which children can maintain a high quality of sleep, a good morning-type diurnal rhythm, and indirectly good mental health [16,17]. The mechanism involves metabolism of tryptophan in the morning to serotonin, a natural anti-depressant, and subsequent conversion of serotonin at night in the pineal gland to melatonin, a natural sleep-onset agent. Consequently, it is possible that inadequate intake of tryptophan at breakfast could affect student’s daytime academic performance and sleep quality.

Eating breakfast can be defined in various ways, including what a person perceives as breakfast, the type of food consumed, a meal consumed at a specified time of day, or the first meal consumed after awakening [23]. In the Inner Mongolia Medical College, breakfast is supplied by college collective canteens, and the type of food consumed is relatively constant, similar to most Chinese collective canteens. Typically, the traditional Chinese breakfast consists of varieties of congee and pickle, soybean milk, fritters, buns, and noodles. We chose the Student Nutrition Dietary Assessment’s definition of breakfast for this study.

Our study shows that the prevalence of regular breakfast consumption is lower compared to the recently reported prevalence among medical students in western countries: for example 80% in the United States [24], 90% in France [25], and 72.5% in Australia [26]; however, it is higher compared to Lebanon (52.7%). It is noted that the prevalence of eating breakfast regularly for male students in our study (52.1%) is roughly equivalent to male Lebanese medical students (58.3%) [27], a prevalence close to the lowest value which we found from the relevant literature. In contrast, regular breakfast consumption for Inner Mongolia Medical College students was lower than that reported in other areas of China: 83.6% for Beijing Medical University and Kunming Medical College [12]. On the other hand, breakfast consumption habits varied considerably according to different population lifestyle habits. Thus, the prevalence of eating breakfast regularly for male students in their second year of education was 49.7%, while for male students in their fourth year of education it was 54.3%. In particular, it was observed that the prevalence of regular breakfast consumption for male students who had >1000 yuan monthly expenses was only 57.3%. This breakfast consumption prevalence is lower than the lowest value we found from the relevant literature [27]. Apparently, that many medical students do not eat breakfast on a regular basis is a very serious public health problem.

Our study also showed that regular breakfast consumption among men was significantly lower compared to women. This finding confirms the gender difference in regard to eating breakfast on a regular basis as reported in a number of recent studies [24,26,28,29], and suggests that there should be a stronger focus among males in regarding to encouraging better breakfast consumption habits*.*

We did not find that the prevalence of breakfast consumption differed among various ethnic groups even though Inner Mongolia is considered one of China’s five minority areas. We speculate that the reason for this result could be because these ethnic groups have resided in the area a long time and have formed similar breakfast eating habits.

Our research results also showed that students who had higher monthly expenses were more likely to skip breakfast than those who had lower monthly expenses. Clearly, multiple logistic regression analysis demonstrated that higher monthly expenses are a risk factor in regard to medical students’ breakfast consumption habits*.* Furthermore, the results of the linear-by-linear association chi square test support an increasing linear trend for the prevalence of eating breakfast regularly as monthly expenses decrease. One possibility could be that students who have higher monthly expenses eat more snacks later in the day compared to those who have lower monthly expenses. In China, fried foods and soft drinks have emerged as the preferred type of snack, which also contain excessive energy and fat [30]. Recent studies have also observed that snackers generally have a higher energy intake compared to non-snackers and that the increased energy intake of adolescents and young adults is mainly attributable to the population who increase their energy intake from snacking [31]. Another important result from these studies is that snackers do not have the feeling of hunger in the morning, which is why they skip breakfast. Additionally, it has been found that small changes in diet composition have the potential to affect feelings of hunger and satiety [19] and that when daily nutrition is not satisfied, people tend to become obese [11]. Most importantly, those individuals who skip breakfast are more likely to be obese compared to students who eat breakfast regularly [32]. Thus, school administrators should consider formulating appropriate educational interventions to help students recognize that the provision of energy and nutrients through the eating of breakfast positively affects energy and nutrient intake throughout the day.

We also noted that the prevalence of regular breakfast consumption varied considerably according to the faculty in which the student belonged: the prevalence of skipping breakfast for students studying in Traditional Chinese Medicine and Mongolian Medicine (22.7%) was much lower than for other faculties, with the highest for the Public Administration and Information Management faculty (35.0%). This result might be explained by the health concepts embodied in traditional Chinese medicine, which emphasizes the importance of breakfast. This situation should be addressed as students in these faculties are not only future public health managers, but may be involved in formulating breakfast promotion education.

The prevalence of regular breakfast consumption in medical students who had a good self-perception about sleeping quality was significantly higher than for students with a bad self-perception. Moreover, there was a dose response relationship for this factor with the prevalence of regular breakfast consumption increasing with better perception of sleep quality. Although poor sleep might result in difficulty in getting up and less time to eat breakfast, one study suggests that poor-quality sleep is associated with an increased risk of poor appetite [33], which may be a direct cause for lack of nutrients and energy in this population. These findings indicate that public health managers should pay more attention to medical students’ sleeping quality, and that research should be conducted to determine the reasons for sleeping poorly.

Our study also showed that the prevalence of regular breakfast consumption in medical students who perceived that learning was easy was much higher compared with their classmates who perceived learning to be laborious. This factor also demonstrated dose dependency. Other studies have confirmed these results. For example, missing breakfast is associated with adverse effects on academic performance and school attendance in young people [6]. We surmise that this is a vicious circle in which poor academic performance and school attendance could cause this group of students to skip breakfast, and that this poor dietary habit could cause poor academic performance and school attendance. It has been proposed that appropriate manipulation of the school environment could offer an efficient and effective long-term means of improving the health of the population [5], and that such an intervention could bring about improved breakfast habits resulting in improved cognitive functioning and behavior, and thus improved school performance and educational achievement [5]. In any case, making students aware of this problem should be a high priority.

The results of the linear-by-linear association chi square test showed no linear trend in regard to class years of education although binary logistic regression analysis demonstrated that class years of education is significantly associated with breakfast consumption habits. The prevalence of regular breakfast consumption in the first and fifth class years of education was significantly higher than in years 2–4, suggesting that students are at risk in this age group. While students in their first year might still be adhering to good family breakfast-eating habits they learned prior to entering college, the explanation for fifth-year students is less obvious. One possible reason might be that their class load in the morning is less so they might have more time to eat breakfast. However, as one study of Chinese university students concluded it may have more to do with getting up sufficiently early in the morning that there is time for breakfast [10]. To address this situation, we propose to examine the feasibility and value of a stepped wedge and long-term cluster randomized trial to evaluate pragmatic public health intervention programs [34] for this group so that changes in attitude and behavior toward breakfast can be tracked.

## Conclusions

The prevalence of regular breakfast consumption in relation to different populations has a large variance. Gender, class years of education, monthly expenses, faculty, appetite, sleep quality, and the learning process were found amongst others to be significant factors associated with regular breakfast eating among medical students in the Inner Mongolia area of China. In addition, monthly expenses, sleep quality, and the learning process showed a dose-dependent relationship. Our study findings could help health care professionals develop targeted interventions designed to increase breakfast consumption.

## Competing interests

We have no competing interests.

## Authors' contributions

The work presented here was carried out in collaboration between all authors. JS and HY defined the research theme and methods. ZL and YW designed the questionnaire, analyzed the data, interpreted the results, and wrote the paper. JB, YYW and YE co-worked on associated data collection and their interpretation. GL and WH discussed analyses, interpretation, and presentation. TW and HL carried out the survey and also helped write the paper. All authors have contributed to, seen, and approved the manuscript.

## Pre-publication history

The pre-publication history for this paper can be accessed here:

http://www.biomedcentral.com/1471-2458/13/42/prepub
